# Biogenic ZnO-CuO Nanocomposites Synthesised Using *Salvia africana* Luteus Increased the Radiosensitising Effect of Proton Irradiation in MCF7 Breast Cancer Cells

**DOI:** 10.3390/nano16130789

**Published:** 2026-06-23

**Authors:** Kunle Okaiyeto, Bartosz Klebowski, Susi Zara, Maria Rosa Gigliobianco, Piera Di Martino

**Affiliations:** 1 Department of Pharmacy, University “G. d’Annunzio” Chieti-Pescara, Via dei Vestini 31, 66100 Chieti, Italy; okaiyeto.kunle@unich.it (K.O.); susi.zara@unich.it (S.Z.); piera.dimartino@unich.it (P.D.M.); 2 Institute of Nuclear Physics, Polish Academy of Sciences, 31-342 Krakow, Poland; bartosz.klebowski@gmail.com

**Keywords:** *Salvia africana* Luteus, green synthesis, biogenic nanoparticles, radiosensitizers, radiotherapy, holotomographic technology

## Abstract

Radiation therapy is widely used for cancer treatment. To improve therapeutic efficacy, traditional radiosensitizers are often used in combination. However, their toxic side effects necessitate urgent development of safer alternative biogenic radiosensitizers. Herein, a green approach was used to synthesise ZnO NPs, CuO NPs, and ZnO-CuO NCs using *S. africana* Luteus, and their ability to enhance the radiosensitizing effect of proton irradiation on Michigan Cancer Foundation-7 (MCF7) breast cancer cell line was evaluated. The biogenic nanoparticles are characterised in detail through several analytical techniques, including Ultraviolet-visible (UV-Vis) spectroscopy, X-ray diffraction (XRD), Fourier Transform Infrared (FTIR) spectroscopy, and Scanning Electron Microscopy (SEM). Interestingly, the NPs showed concentration-dependent effects on MCF7 viability, with CuO NPs exhibiting the strongest effect (IC_50_ = 42.90 µg/mL), followed by ZnO-CuO NCs (71.12 µg/mL) and ZnO NPs (103.43 µg/mL). Proton irradiation produced a dose-dependent decrease in clonogenic survival of MCF7 cells, and ZnO-CuO NCs displayed the highest enhancement of proton-induced cell death, with a Dose Enhancement Factor (DEF) of 1.69, compared with CuO NPs (1.46) and ZnO NPs (1.09). Holotomographic microscopy (HTM) data further confirmed that ZnO-CuO NCs impaired cellular macromolecules more than the individual NPs. Findings from this study suggest that the biogenic NPs are promising radiosensitizers for cancer radiotherapy.

## 1. Introduction

Drug resistance is a major challenge in treating various cancers, including breast cancer, which is one of the leading causes of cancer-related mortality in women globally [1]. Rising resistance to conventional chemotherapy, coupled with severe, often life-threatening adverse effects, has prompted an urgent need for safer treatment approaches [2]. Radiation therapy is widely used to treat cancer in developed countries worldwide [3]. Despite its effectiveness, radiation cannot differentiate between cancer cells and healthy cells. Therefore, precision radiation is needed to circumvent this problem. Notably, synthetic radiosensitizers such as 5-fluorouracil, gemcitabine, and cisplatin are often used in combination with radiation to enhance cancer cell sensitivity to radiotherapy [4]. Although these radiosensitizers have demonstrated significant advances in oncology, considerably improving survival rates for patients with various cancer types, especially those resistant to conventional radiotherapy, they work by enhancing radiation’s ability to kill cancer cells, often by disrupting deoxyribonucleic acid (DNA) repair mechanisms or reducing tumour hypoxia [5]. These agents allow for higher therapeutic ratios in difficult-to-treat cases. Nonetheless, the adverse side effects associated with conventional radiosensitizers cannot be ignored, as they directly affect a patient’s quality of life and treatment continuity. As a result, there is an urgent need to develop safer alternatives, such as nanotechnology-based approaches, to produce biogenic radiosensitizers that are more effective, cause fewer toxic effects, and yield improved patient outcomes.

Nanotechnology involves manipulating matter at the atomic and molecular levels, typically between 1 and 100 nanometers, to produce materials with unique, improved, or entirely new chemical and physical characteristics [6]. Remarkably, nanotechnology has continued to receive significant global attention due to its cutting-edge nature and wide-ranging applications across various fields, especially in biomedical sciences. Recent advances in nanotechnology have transformed biomedical research and provided considerable relief in oncology research. There are three methods of synthesising NPs. Conventional physical methods rely on sophisticated, expensive, and labour-intensive machines, whereas chemical methods employ toxic, environmentally harmful reagents; these limitations limit their applicability in medicine. Therefore, a search for an alternative, safer approach to the synthesis of NPs has been a major focus in biotechnology, biomedicine, and pharmaceutical technology. As a sequel to this, green nanotechnology has been identified as a safer, non-toxic, and eco-friendly alternative with therapeutic efficacy. In addition, this method is associated with reduced processing time and ease of handling [7]. In green synthesis of NPs, phytochemical compounds present in plants serve as reducing and stabilising agents [8]. Among metal oxide NPs, ZnO NPs and CuO NPs have gained attention in biotechnology and pharmaceutical technology [9], owing to their physicochemical properties, including small size, large surface area-to-volume ratio, wide band gap, optical properties, high thermal and chemical stability, biocompatibility, and high biological activity [10].

The genus Salvia (Lamiaceae) comprises approximately 900 species worldwide, with a few species native to Africa, including *Salvia africana* Lutea [11,12]. The name “Salvia” derives from the Latin “salvare,” meaning “healer.” These plant species are used for ornamental purposes, aromatherapy, and the treatment of various illnesses. *S. africana* Lutea, also referred to as beach sage or Bruinsalie, is widely distributed in the Eastern Cape Province of South Africa. The conservation status of *S. africana* Lutea is classified as “least concern” due to its abundance, broad distribution, and low risk of extinction. The plants have demonstrated exceptional tolerance to drought or water scarcity and thrive in sandy and coastal regions. These hardy plants have distinctive clusters of brown funnel-shaped flowers and fragrant grey-greenish foliage [11]. *S. africana* Lutea is locally used in treating different infections or diseases, mental and nervous disorders, tuberculosis, chronic bronchitis, diarrhoea, stomach aches, throat inflammation, etc. [13]. A few medicinal properties, including antibacterial [14], antifungal [15], anticancer [16], and antidiabetic [13], of *S. africana* Luteus have been reported in the literature. Despite the multipurpose folkloric uses of this plant in treating several human ailments, only a few scientific studies have been documented in the literature to date. From a bibliometric perspective on research on the Salvia genus, only 0.23% of studies are related to *S. africana* Lutea, indicating that the plant is still underutilised [11]. A previous study by Ezema et al. [11] has highlighted that the biological activities of *S. africana* Lutea could be due to the presence of phytochemical compounds including, “rosmarinic acid, salvianolic acid K, rosmadial, rosmarinic acid, salvianolic acid B, 2,4-dimethyl benzenepropanoic acid, quinic acid, danshensu, caffeoyl acid, yunnaneic acid E (isomer 1–6), hydroxy-luteolin–glucuronide, yunnaneic acid F, yunnaneic acid D (isomer 1–2), luteolin-7-O-glucuronide, sagerinic acid (isomer 1), caffeoyl, rosmarinic acid (isomer 1–3), thymol, carvacrol, and (Z)-isoeugenol”.

A previous study has explored *S. africana*-Lutea for synthesising silver and gold nanoparticles and has evaluated their antibacterial activity against *Staphylococcus epidermidis* and *Pseudomonas aeruginosa* [17]. Nonetheless, to the best of our knowledge, this is the first study reporting the synthesis of biogenic ZnO NPs, CuO NPs, and ZnO-CuO NCs using an aqueous extract of *S. africana* Luteus, evaluating their potential to inhibit the viability of MCF7 breast cancer cells in an in vitro model, and investigating their ability to increase the sensitivity of MCF7 cells to proton irradiation. To the best of our knowledge, this is the first study reporting the radiosensitivity of biogenic NPs produced from *S. africana* Luteus. The development of a green radiosensitizer will significantly improve the efficacy of cancer radiation therapy by increasing bioavailability, reducing harmful effects on healthy tissues, and boosting therapeutic outcomes; thus, improving the patient’s quality of life and treatment continuation while overcoming the problems associated with the use of conventional radiosensitizers.

## 2. Materials and Methods

### 2.1. Plant Collection and Extraction

*S. africana* Luteus was harvested from the plant garden of Università degli Studi “G. d’Annunzio” Chieti—Pescara, Italy, and the leaves were harvested and washed three times with distilled water to remove any dust particles. The leaves were air-dried in the shade for 2 weeks and pulverised with a blender to make a powder. Thereafter, 10 g of the plant powder was weighed and transferred into a 500 mL beaker containing 200 mL of distilled water, which was later placed on a magnetic stirrer (Velp Scientifica srl, Usmate, Italy) and heated at 80 °C for 1 h under continuous stirring at 600 rpm. Subsequently, the mixture was allowed to cool down to room temperature and then filtered with Whatman filter paper No. 1, followed by centrifugation at 6000 rpm for 30 min. The clear filtrate was transferred into clean Falcon tubes and stored in the refrigerator at 4 °C for green synthesis.

### 2.2. Green Synthesis

The green synthesis of ZnO NPs and CuO NPs was conducted as described by Adeyemi et al. [17,18] with some modifications. Briefly, 50 mL of aqueous extract of *S. africana* Luteus was mixed with 450 mL of 0.1 M zinc acetate dihydrate (Zn(CH_3_CO_2_)_2_·2H_2_O), or 0.1 M copper (II) sulfate pentahydrate (CuSO_4_‧5H_2_O) in a clean 1 L beaker, and the mixture was adjusted to pH 10 with 2 M NaOH. Each of the mixtures was tightly covered with aluminium foil and placed on a magnetic stirrer separately and heated at 85 °C for 3 h under constant stirring at 600 rpm. Thereafter, the suspended mixture was centrifuged at 6000 rpm for 30 min, and later, the supernatant was discarded, and the precipitate (biogenic ZnO NPs or CuO NPs) was resuspended in distilled water and washed four times by repeating the same process. The recovered biogenic ZnO NPs and CuO NPs were dried in an oven (Ostba GG-801, Paris, France) overnight at 60 °C, and calcinated (Muffle furnace 8.2 lt-FM 8.2, Treviglio, Italy) in a furnace at 400 °C for 2 h, and the final products were stored in airtight containers in the fridge at 4 °C until use.

For the synthesis of ZnO-CuO NCs, 50 mL of aqueous extract of *S. africana* Luteus was mixed with 225 mL of 0.1 M zinc acetate dihydrate Zn(CH_3_CO_2_)_2_·2H_2_O, and 225 mL of 0.1 M copper (II) sulfate pentahydrate (CuSO_4_‧5H_2_O) in a 1 L beaker, and the mixture was adjusted to pH 10 with 2 M NaOH. The beaker containing the mixture was tightly covered with aluminium foil and placed on a magnetic stirrer and heated at 85 °C for 3 h. Subsequently, the suspended mixture was centrifuged at 6000 rpm for 30 min, with the supernatant discarded and the precipitate (ZnO-CuO NCs) reconstituted in distilled water and washed four times. The recovered ZnO-CuO NCs were dried in an oven (Ostba GG-801, Paris, France) overnight at 60 °C and calcinated in a furnace (Muffle furnace 8.2 lt -FM 8.2, Treviglio, Italy) at 400 °C for 2 h and stored in an airtight container in the refrigerator at 4 °C until use.

### 2.3. Characterisation of Biogenic Nanoparticles

UV-Vis spectroscopy analysis (Multiskan™ SkyHigh Spectrophotometer, Thermo Scientific, Singapore) was used to confirm the synthesised ZnO NPs, CuO NPs, and ZnO-CuO NCs from *S. africana* Luteus in the range of 200–800 nm. The average particle size and PDI were determined by the Dynamic Light Scattering (DLS) technique using a Zetasizer Advanced (Malvern Panalytical, model: 10019524, Worcestershire, UK). The PDI provides a measure of particle size distribution, with values approaching 0 indicating monodisperse populations and values above 0.5 indicating heterogeneity. Measurements were performed on NPs re-dispersed in deionized water at 25 °C, sonicated for 15 min, using a backscattering angle of 173°, using a 632.8 nm laser diode with a rated output of 10 mV. ζ-Potential was measured with the same instrument using a capillary zeta cell at 25 °C. All experiments were conducted in triplicate, and results are reported as mean ± standard deviation. To identify the functional groups of the phytochemical compounds involved in the reduction and stabilisation of the synthesised NPs, the FTIR analysis was conducted using a Shimadzu IR Affinity-1S FTIR spectrophotometer (Shimadzu Italia S.r.l., Milan, Italy) equipped with a sealed and desiccated interferometer, a DLATGS (Deuterated Triglycine Sulphate Doped with L-Alanine) detector, and a single reflection diamond Attenuated Total Reflectance (ATR) crystal (QATR 10, Shimadzu Italia S.r.l., Milan, Italy). All spectra were collected with 45 scans over a range of 4000 to 400 cm^−1^ at 25 °C, with a resolution of 4 cm^−1^, using Happ–Genzel apodization. Furthermore, the surface morphology, shape, and size of the synthesised NPs were studied using SEM coupled with EDX using Phenom XL G2 Benchtop SEM (Thermo Fisher Scientific, Brno, Czech Republic) to determine their elemental compositions. The size and morphology of the NPs were determined by transmission electron microscopy (TEM) in scanning transmission electron microscopy (STEM) mode using an FEI Talos transmission electron microscope (Thermo Fisher Scientific, Waltham, MA, USA). The purity and crystallinity of the synthesised NPs were confirmed by X-ray Diffraction (XRD) analysis using an X-ray diffractometer (Bruker D2 Phaser, Bruker, Karlsruhe, Germany) in the 2θ range of 20–80° at 40 kV and 40 mA with a scanning speed of 4°/min.

The crystallite size was calculated using the Debye-Scherrer Equation (1):

D = Kλ/(βCosθ)(1)

where D is the nanoparticle crystalline size, K is the Scherrer constant (0.9), λ is the wavelength (1.54), and β is the full width at half maximum (FWHM).

### 2.4. Cell Culture

MCF7 breast cancer cells were purchased from the American Type Culture Collection (ATCC, Manassas, VA, USA). MCF7 cells were cultured in Dulbecco’s Modified Eagle Medium (DMEM) high-glucose medium supplemented with 10% foetal bovine serum (FBS) and 1% antibiotic mixture (5000 units/mL penicillin, 5 mg/mL streptomycin, and 10 mg/mL neomycin). The cells were cultured by biweekly passages in a 37 °C humidified atmosphere with 5% CO_2_.

### 2.5. MTS Assay

The effect of ZnO NPs, CuO NPs, and ZnO-CuO NCs on MCF7 cell viability was determined using 3-(4,5-dimethylthiazol-2-yl)-5-(3-carboxymethoxyphenyl)-2-(4-sulfophenyl)-2H-tetrazolium (MTS) assay (CellTiter 96^®^ Aqueous One Solution Cell Proliferation Assay, Promega, Madison, WI, USA). For this purpose, MCF7 cells were seeded into 96-well plates at a density of 10^4^ cells/well in DMEM containing 10% FBS. After 24 h, the medium was replaced with a fresh one, which additionally contained NPs at concentrations ranging from 5 to 150 µg/mL. MCF7 cells cultured in medium without NPs served as a control. For each experimental condition, cells were analysed in three technical replicates (three wells per condition). The entire experiment was independently repeated three times using separately cultured cells, resulting in three biological replicates (*n* = 3). The cells were incubated with the NPs for 24 h. After incubation, the medium was replaced with fresh medium (100 µL), followed by the addition of 20 µL of MTS reagent to each well. After 2 h of incubation, cell viability was measured using a Multiskan SkyHigh Plate Reader (Thermofisher, Waltham, MA, USA) by recording the absorbance at 490 nm. Cell viability was estimated as the ratio of the absorbance of cells exposed to NPs to the absorbance of control cells, represented by cells receiving only medium. Finally, IC_50_ values were estimated through the MTS assay using a four-parameter logistic model.

### 2.6. Proton Irradiation Protocol

Proton irradiation was conducted using a Proteus C-235 (Ion Beam Applications, Louvain-la-Neuve, Belgium) isochronous cyclotron located at the Cyclotron Centre, Bronowice. Irradiations were performed at room temperature with doses of 1, 2, 5, and 10 Gy. The pencil-beam scanning technique was applied. A monoenergetic proton field with an energy of 225 MeV and a field size of 20 cm × 20 cm was used. Irradiations were carried out at a water-equivalent depth of 1.1 cm, achieved using a 1 cm RW3 phantom plate. Petri dishes containing the cell cultures were positioned centrally on the beam axis at the isocenter and irradiated vertically from below (gantry angle 180°). Before irradiation, dosimetric measurements were performed using a Markus-type ionisation chamber calibrated in terms of absorbed dose to water.

### 2.7. Clonogenic Assay

MCF7 cells were trypsinised and seeded into Petri dishes at different densities, depending on the stringency of proton irradiation (ranging from 500 to 2500 cells per Petri dish). After 24 h of incubation, the culture medium was replaced with fresh medium supplemented with ZnO NPs, CuO NPs, or ZnO-CuO NCs at a concentration of 25 µg/mL. The concentration of 25 µg/mL was selected based on the MTS viability assay as a well-tolerated dose for all tested NPs, ensuring comparable experimental conditions and allowing the evaluation of radiosensitisation effects independent of direct NPs-induced toxicity. After an additional 4 h, the cells were exposed to proton beam irradiation at doses of 1, 2, 5, and 10 Gy. Following irradiation, the cells were incubated for two weeks to allow colony formation. Subsequently, the colonies were fixed with 5% formaldehyde, rinsed with distilled water, and stained with 1% crystal violet. Colonies consisting of viable cells were then counted. The plating efficiency (PE) was calculated as the ratio of the number of colonies formed to the number of cells seeded. The surviving fraction (SF) was determined as the ratio of the PE of irradiated cells to the PE of the corresponding non-irradiated control. Survival curves (SF versus dose) were plotted to illustrate the results of the assay. Finally, based on the clonogenic assay results, the quantitative dose enhancement factor (DEF) at a surviving fraction of 0.1 (SF 0.1) was calculated in accordance with the report of Subiel et al. [18,19].

### 2.8. Holotomographic Imaging

The holotomographic imaging was performed using Nanolive 3D Cell Explorer-Fluo (Tolochenaz, Switzerland) microscope equipped with a ×60 magnification objective, λ = 520 nm, sample exposure 0.2 mW/mm^2^, and field depth of 30 μm. For this experiment, MCF7 cells were seeded into a 96-well plate made of SBS material, enabling real-time imaging of cell-NP interactions. For this purpose, the cells were treated with medium containing ZnO NPs, CuO NPs, or ZnO-CuO NCs at concentrations of 25 µg/mL and 75 µg/mL. Concentrations of 25 and 75 µg/mL were chosen as common non-cytotoxic and cytotoxic doses, respectively, for all NPs. Throughout the entire experiment, the cells were maintained in a microscope incubator providing optimal conditions for their growth. Based on differences in the refractive index (RI), a 3D holotomographic reconstruction was generated, allowing the distinction of individual cellular structures (e.g., cell membrane and cytoplasm) as well as the assessment of NPs localization. Holotomographic images were acquired both before the addition of NPs and after the addition of NPs at different time points (1 h, 2 h 30 min, 4 h, and 24 h).

### 2.9. Statistical Analysis

The MTS and clonogenic assay results were shown as the means ± SEM (standard error of mean) from three independent experiments. Within each biological replicate, all conditions were analysed in triplicate technical replicates. The MTS test data were also analysed using one-way analysis of variance (ANOVA) followed by a post hoc Tukey test. Statistical significance was accepted when *p* < 0.05.

## 3. Results and Discussion

### 3.1. UV-Vis Spectroscopy Analysis

**Figure 1:**
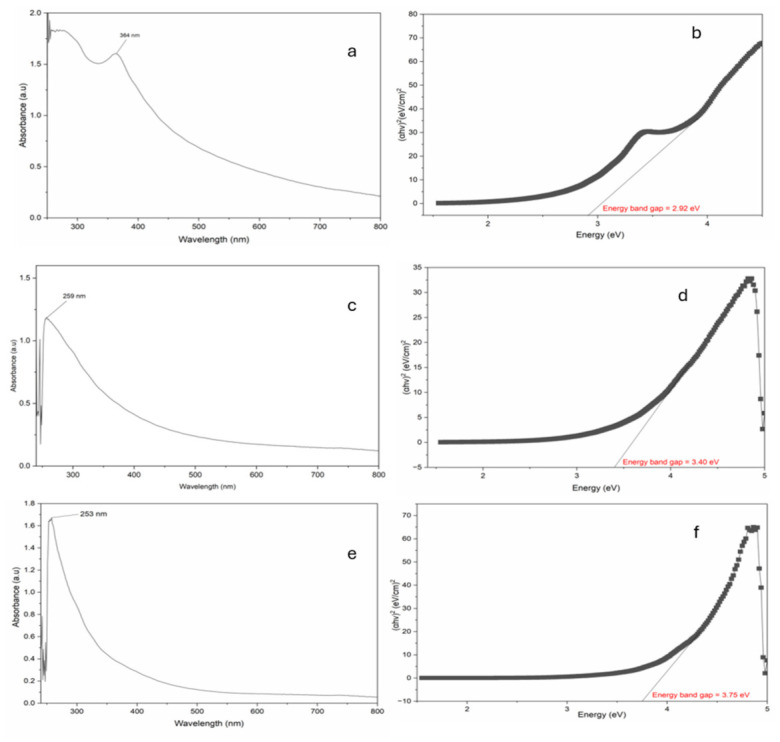
UV-Vis spectroscopy analysis of ZnO NPs (**a**), CuO NPs (**c**), and ZnO-CuO NCs (**e**) and energy band gap of ZnO NPs (**b**), CuO NPs (**d**), and ZnO-CuO NCs (**f**) synthesised using *S. africana* Luteus.

In the present study, the synthesis of ZnO NPs, CuO NPs, and ZnO-CuO NCs was confirmed by UV-Vis spectroscopy analysis. A unique absorption peak at 364 nm was recorded for ZnO NPs (Figure 1a), with an energy band gap of 2.92 eV (Figure 1b). This absorption peak was due to the surface plasmon resonance of the metal oxide NPs in an electromagnetic field that allows the transfer of electrons from the valence band to the conduction band [20]. A study conducted by Brishti et al. [21] reported a strong absorption peak at 326 nm for ZnO NPs synthesised using *Epipremnum aureum* aqueous extract with an energy band gap of 3.73 eV, and their observations substantiate our findings in the present study. In the case of *S. africana* Luteus-mediated CuO NPs, the absorption peak was observed at 259 nm (Figure 1c), with an energy band gap of 3.40 eV (Figure 1d). In agreement with our findings, Labaran et al. [22] reported that the synthesis of CuO NPs from *Alstonia scholaris* displayed at 243 nm. The ZnO-CuO NCs synthesised using *S. africana* Luteus in the present study exhibited an absorption peak at 253 nm (Figure 1e) and a band gap of 3.75 eV (Figure 1f). Endeshaw et al. [22] reported that the ZnO-CuO NCs synthesised using *Croton macrostachyus* were recorded at 375 nm with an energy band gap of 2.68 eV.

### 3.2. DLS Analysis

The hydrodynamic diameter, polydispersity index (PDI), and zeta potential of *S. africana* Luteus-mediated ZnO NPs, CuO NPs, and ZnO-CuO NCs were determined using DLS analysis. The hydrodynamic diameter of ZnO NPs was 357 nm, with a PDI of 0.36 and a zeta potential of −30.78 mV. For the CuO NPs, the hydrodynamic diameter was 174 nm, the PDI was 0.48, and the zeta potential was −11.31 mV, while for the ZnO-CuO NCs, the hydrodynamic diameter was 248 nm, the PDI was 0.22, and the zeta potential was −18.64 mV (Table 1). It has been reported that smaller NPs are more effective for biomedical applications than larger ones due to their high surface-to-volume ratio [23]. Likewise, it is worth highlighting that the particle sizes measured by DLS are usually larger than those measured by other techniques because particles are dispersed in water during analysis, and water molecules surrounding the particles increase the measured hydrodynamic diameter of the particles.

Furthermore, some researchers have highlighted that the strong intermolecular hydrogen bonds between hydroxyl groups of different phenolic compounds can lead to particle aggregation, resulting in larger sizes of the *S. africana* Luteus-mediated ZnO NPs, CuO NPs, and ZnO-CuO NCs observed in the DLS data [24]. The report of Xie et al. [25] has emphasised that NPs with a PDI of less than 0.4 suggest that they are homogeneous and monodispersed in colloidal suspension. The polydispersity index is important for assessing the uniformity of particle behaviour in suspension [26]. Our findings suggest that the three biogenic NPs were monodispersed, which corroborates the reports from previous studies [27,28]. Moreover, the high negative zeta potentials observed for all the synthesised biogenic NPs in the present study indicate strong repulsive forces among particles, which help ensure the stability of the NPs in colloidal suspension [29]. The stability of NPs is greatly influenced by the nature and amount of phytochemical compounds that stabilise the NPs. Therefore, zeta potential is directly influenced by the surface chemistry of the NPs. The zeta potential and size of NPs affect their toxicity to microorganisms [30].

### 3.3. XRD Analysis

**Figure 2:**
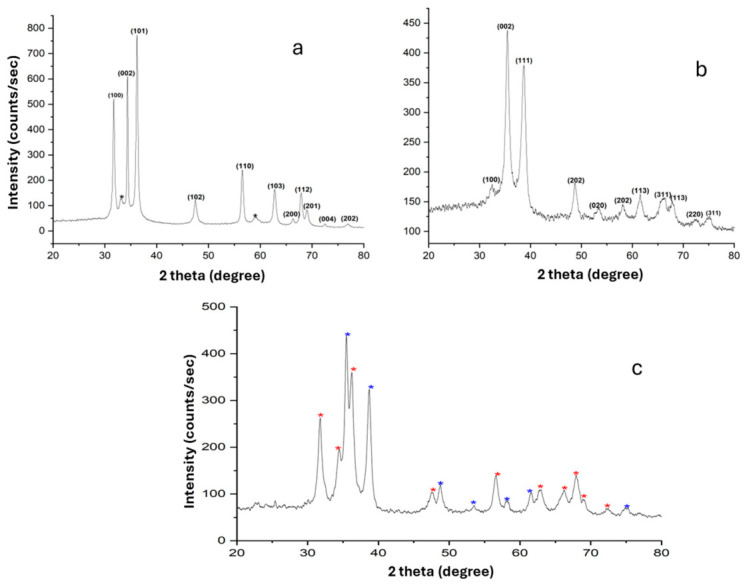
XRD profiles of ZnO NPs (**a**), CuO NPs (**b**), and ZnO-CuO NCs (**c**) synthesised using aqueous extract of *S. africana* Luteus. The red asterisk (*) colour denotes the presence of ZnO NPs, peaks, while the blue indicates CuO NPs.

The formation of the biogenic NPs was also confirmed by XRD analysis [31,32]. Figure 2 reveals the XRD patterns of ZnO NPs, CuO NPs, and ZnO-CuO NCs synthesised using *S. africana* Luteus. The diffraction patterns for ZnO NPs were recorded at 2θ values of 31.73°, 34.38°, 36.22°, 47.49°, 56.56°, 62.81°, 66.45°, 67.91°, 69.03°, 72.56° and 76.58° corresponding to (100), (002), (101), (102), (110), (103), (200), (112), (201), (004) and (202) lattice planes, respectively. This XRD profile revealed a hexagonal wurtzite crystal structure in the *S. africana* Luteus-mediated ZnO NPs, consistent with the JCPDS card number 01-080-0075 [33]. The noticeably high intensity peak at (101) suggests the orientation of the crystallites [33]. The thick bioorganic coating surrounding the NPs may act as a strong crystalline shell, giving rise to sharp Bragg reflections in the XRD pattern. Our findings are consistent with other previous studies documented in the literature [21,34]. Furthermore, the two small peaks that appear on the XRD profile could be due to the amorphous material from the phytochemical compounds stabilising the NPs [35]. The average crystallite size of ZnO NPs was calculated using the Debye–Scherrer formula using eight peaks from the XRD spectra and was found to be 18.54 nm (Table 2).

For CuO NPs, the XRD patterns were observed at 2θ values of 32.81°, 35.48°, 38.64°, 48.77°, 53.09°, 58.35°, 61.54°, 66.25°, 68.04°, 72.25°, and 75.03° corresponding to (110), (002), (111), (202), (020), (202), (113), (311), (113), (220), and (311) lattice planes, respectively [36]. The diffraction peak at 2θ values of 38.7 corresponds to the (111) plane, suggesting the crystalline and monoclinic nature of the synthesised CuO NPs, which matched with JCPDS No. 05–0661 [32]. In the present study, the average crystallite size of CuO NPs was calculated from the eight peaks from the XRD spectra and was found to be 7.89 nm (Table 2).

For ZnO-CuO NCs, the XRD patterns were observed at 2θ values of 31.76°, 34.72°, 35.46°, 36.20°, 38.67°, 47.57°, 48.76°, 53.50°, 56.60°, 58.14°, 61.56°, 62.85°, 66.18°, 67.98°, 69.07°, 72.45°, and 74.96°, respectively [37]. These diffraction peaks matched both ZnO NPs and CuO NPs with no extra peaks, indicating the successful synthesis of pure ZnO-CuO NCs [38,39]. The presence of two distinct phase structures in the NCs suggests the separate formation of CuO NPs and ZnO NPs [32]. Furthermore, the intensity and quality of the peaks for ZnO NPs, CuO NPs, and ZnO-CuO NCs reflect the purity and crystallinity of the biogenic NCs synthesised using *S. africana* Luteus in the present study. In addition, the average crystallite size of the ZnO-CuO NCs was calculated to be 12.91 nm (Table 2). Notably, it is worth mentioning that the average crystalline size of the ZnO-CuO NCs synthesised using *S. africana* Luteus is significantly smaller compared to those reported in previous studies [32,39].

### 3.4. SEM and EDX Analyses

**Figure 3:**
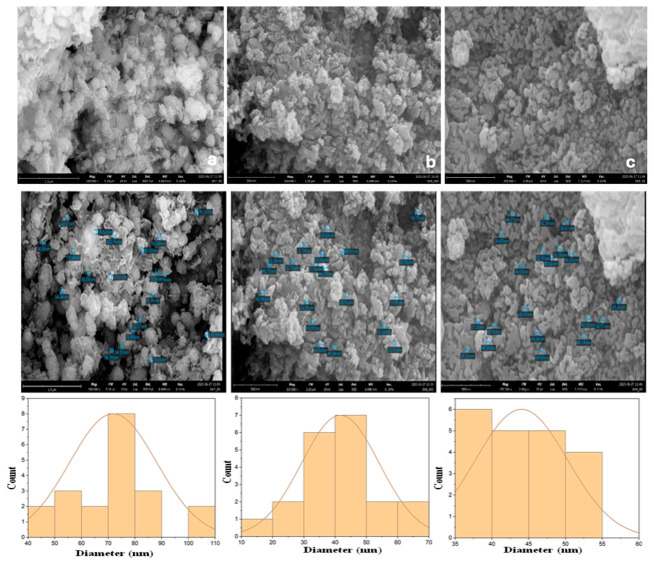
SEM images of ZnO NPs (**a**), CuO NPs (**b**), and ZnO-CuO NCs (**c**) synthesised using aqueous extract of *S. africana* Luteus. The average particle distribution was calculated from the average of 20 measured particles in the SEM images to generate their histograms.

SEM was employed to elucidate the surface morphology, shape, and size of the *S. africana* Luteus-mediated ZnO NPs, CuO-NPs, and ZnO-CuO NCs, and the results are depicted in Figure 3. Notably, these images reveal that the NPs exhibit different morphological structures and aggregations [27]. The image of the biogenic ZnO NPs (Figure 3a) appears as a fluffy, almost spherical, shell-like structure, characterised by agglomeration. The observed agglomeration could be due to several factors, among which are Van der Waals forces of attraction between the phytochemical compounds, most especially the hydroxyl groups of phenolic compounds forming intermolecular forces among themselves on the surfaces of the NPs moving in the colloidal suspension [40]. Van der Waals forces of attraction are primary, short-range, intermolecular forces that drive particle aggregation. Because green-synthesised NPs are often designed to be small with high surface-area-to-volume ratios, these attractive forces can be quite strong, necessitating stabilisation by natural capping agents present in *S. africana* Luteus. The ZnO NPs have a particle size distribution ranging from 40 to 110 nm, with an average size of 71.14 nm. Some researchers have highlighted that small size, increased surface area, and the presence of phytochemical compounds on the surface of the biogenic NPs are the factors influencing the aggregation [32,41].

Furthermore, the SEM image of *S. africana* Luteus-mediated CuO NPs exhibited irregular shapes, comprising both rod-like and spherical shapes, with some agglomeration (Figure 3b). The different shapes observed in the SEM images show the complex and diverse nature of the synthesised NPs, which might influence their physicochemical properties and biomedical applications [22]. The report of Prathap et al. [36] emphasised that the agglomeration in the biosynthesised *Indigofera linnaei* Ali-mediated CuO NPs could be due to high surface energy, among other factors previously highlighted. The biogenic CuO NPs in the present study have a particle size distribution between 10 and 70 nm, with an average diameter measured to be 38.41 nm. Likewise, the morphology of ZnO-CuO NCs was studied, and Figure 3c shows a rod-like shape, like a tube, and some spherical-shaped particles with aggregations, which could be due to the phytochemical compounds on the surface of the NPs stabilising them. The particles have a size distribution between 35 and 55 nm with an average diameter of 43.56 nm.

**Figure 4:**
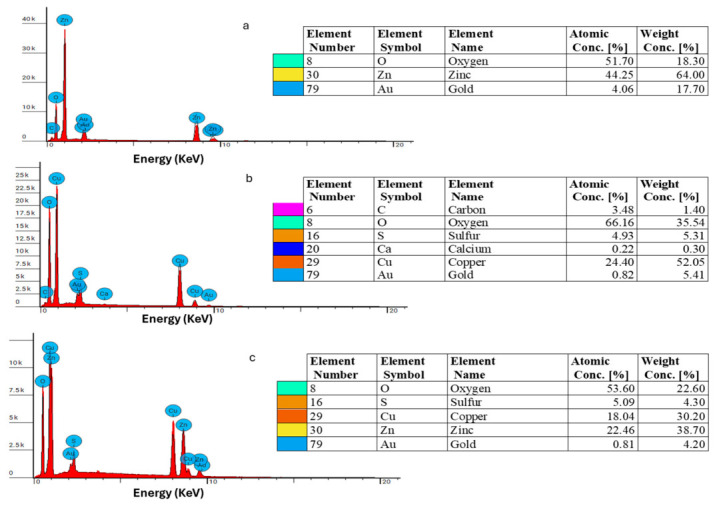
EDX of ZnO NPs (**a**), CuO NPs (**b**), and ZnO-CuO NCs (**c**) nanocomposites synthesised using aqueous extract of *S. africana* Luteus.

Furthermore, EDX analysis was conducted alongside SEM, and this was employed to investigate the elemental compositions of the biogenic nanoparticles and also to confirm their purity. Figure 4 represents the results of the EDX spectra, and it was revealed that the atomic concentration of O was 51.70%, Zn was 44.25%, and Au was 4.06%, while the weight concentration of O was 18.17%, Zn was 64.0%, and Au was 17.70% in the synthesised ZnO NPs. Strong emission peaks of Zn were detected at ~1 keV, 8.6 keV, and 9.6 keV, which agree with earlier studies [42]. Also, for the CuO NPs, the atomic concentration of C was 3.48%, and O was 66.16%, S was 4.93%, Ca was 0.22%, Cu was 24.40%, and Au was 0.82%, whereas the weight concentration of C was 1.14%, O was 35.45%, S was 5.31%, Ca was 0.3%, Cu was 52.05% and Au was 5.41%. Likewise, for the ZnO-CuO NCs, the atomic concentration of O was 53.60%, S was 5.09%, Cu was 18.04%, Zn was 22.46%, and Au was 0.81%, while the weight concentration of O was 22.60%, S was 4.30%, Cu was 30.20%, Zn was 38.7%, and Au was 4.2%. The presence of Au observed in the three synthesised nanoparticles may be due to Au being used to coat the samples before analysis. Our findings in this study are in line with some previously reported studies [27,37,43].

### 3.5. FTIR Analysis

**Figure 5:**
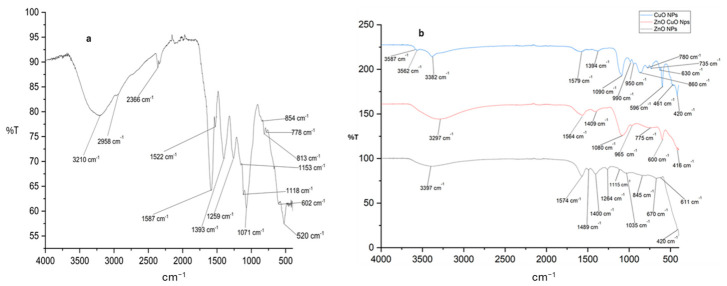
FTIR patterns of aqueous extract of *S. africana* Luteus (**a**) and FTIR patterns of ZnO NPs, CuO NPs, and ZnO-CuO NCs synthesised using aqueous extract of *S. africana* Luteus (**b**).

*S. africana* luteus contains a lot of phytochemical compounds, including polyphenolic compounds and flavonoids, which have the capacity to reduce metal salts into their metallic oxide nanoparticles as well as stabilise the synthesised nanoparticles. This means they have the capacity to perform dual functions (as reducing and stabilising agents). Figure 5a depicts the FTIR spectrum of *S. africana* extract represented different functional groups, including -OH, -CO, -C=H, and -C-H that appear at 3210 cm^−1^, 2958 cm^−1^, 2366 cm^−1^, 1522 cm^−1^, 1587 cm^−1^, 1393 cm^−1^, 1259 cm^−1^, 1153 cm^−1^, 1118 cm^−1^, 1071 cm^−1^, 854 cm^−1^, 813 cm^−1^, 778 cm^−1^, 602 cm^−1^ and 520 cm^−1^. The functional groups of the phytochemical compounds were also detected on the surface of the *S. africana* Luteus-synthesised ZnO NPs, CuO NPs, and ZnO-CuO NCs, since they stabilised them without additional synthetic stabilising agents. Figure 5b depicts the FTIR of ZnO NPs, CuO NPs, and ZnO-CuO NCs synthesised using *S. africana* Luteus, which were investigated within 4000–400 cm^−1^ to determine the functional groups of the phytochemical compounds that are responsible for capping and stabilisation of the synthesised biogenic NPs [44]. In Figure 5, the broad spectrum of hydroxyl groups of phenolic compounds on the surface of ZnO NPs was recorded at 3397 cm^−1^ [45]; the peak at 1574 cm^−1^ suggested (C-C) aromatic stretching, 1489 cm^−1^ and 1400 cm^−1^, suggested (C-H) of alkenes, 1264 cm^−1^, 1115 cm^−1^, 1035 cm^−1^ suggested (C-O) stretching wings of alcohols/carboxylic, 845 cm^−1^, and 670 cm^−1^ indicate the presence of a mono or disubstituted benzene ring (phenyl group). The peak at 670 to 420 cm^−1^ indicated the Zn-O vibration stretching, and our finding is consistent with previous studies [43,45]. Similarly, the FTIR spectra of CuO NPs, with multiple vibrational peaks at 3587 cm^−1^, 3562 cm^−1^, and 3382 cm^−1^, confirmed the presence of hydroxyl groups from diverse phenolic compounds in *S. africana* Luteus, which stabilise the synthesised CuO NPs [46]. The peaks at 1579 cm^−1^ can be assigned to the aromatic bending of the alkene group (C=C) [47]. Also, the peaks at 1394 cm^−1^, 1090 cm^−1^, 990 cm^−1^, and 950 cm^−1^ confirm the presence of the carbonyl group, while the sharp peaks of Cu-O vibration were recorded at 596 cm^−1^, 461 cm^−1^, and 420 cm^−1^ [48]. For the ZnO-CuO NCs, the vibration peak at 3297 cm^−1^ represents the stretch of the hydroxyl group of phenolic compounds [49]. The peaks at 1564 cm^−1^, 1409 cm^−1^, 1080 cm^−1^, 965 cm^−1^, and 775 cm^−1^ represent the stretching of the carbonyl group, while metal-oxygen vibration was confirmed at 600 cm^−1^ and 416 cm^−1^ [50,51]. As highlighted in previous studies, phenolic and flavonoid compounds in *S. africana* Luteus act as capping and stabilising agents to prevent nanoparticle aggregation and further growth [51,52]. Through the release of a reactive hydrogen atom, the flavonoid biomolecules converted the enol form to the keto form, which in turn reduced Zn^2+^ and Cu^2+^ ions to their corresponding ZnO NPs and CuO NPs [47].

### 3.6. TEM Analysis

**Figure 6:**
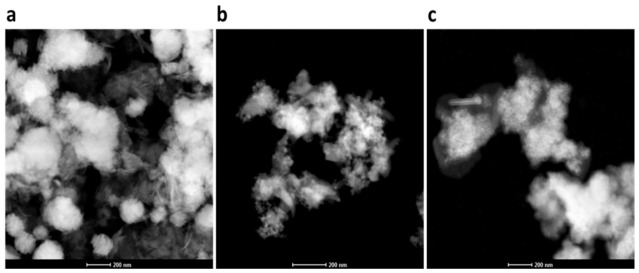
STEM high-angle annular dark-field (HAADF) images of ZnO NPs (**a**), CuO NPs (**b**), and ZnO-CuO NCs (**c**).

Figure 6 depicts the TEM images of the synthesised ZnO NPs, CuO NPs, and ZnO-CuO NCs using *S. africana* Luteus. The images show that the NPs were spherical to irregular shapes, similar to the morphology obtained from SEM images. It is worth highlighting that the TEM images are of higher resolution, providing information that the nanoparticles were separated by interparticle distance and are not in physical contact with one another [53]. The average diameter of ZnO NPs, CuO NPs, and ZnO-CuO NPs was found to be approximately ~214 nm, ~133 nm, and ~197 nm, respectively. Our findings are closely similar to the reports of other researchers [54,55,56].

### 3.7. Effect on Cell Viability and Radiosensitizing Properties of ZnO NPs, CuO NPs, and ZnO-CuO NCs

**Figure 7:**
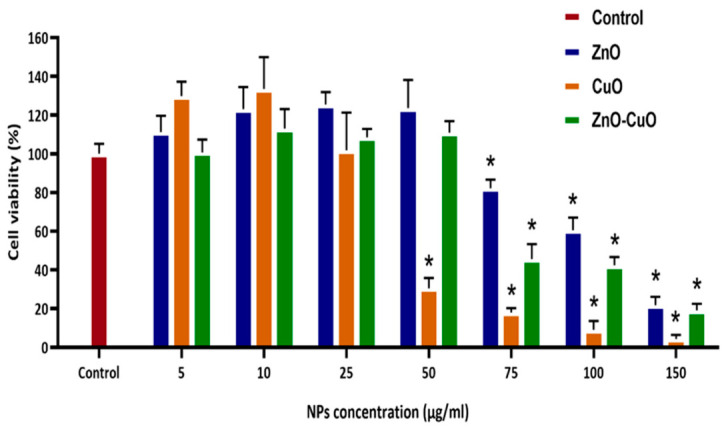
MTS viability test results for MCF7 cells cultured for 24 h with ZnO, CuO, and ZnO-CuO NCs from *Salvia africana* Luteus. Data are presented as mean ± SD from three independent biological experiments (n = 3). * indicates *p* < 0.05 vs. control.

The MTS assay was used to evaluate the concentration-dependent effect on cell viability of ZnO NPs, CuO NPs, and ZnO-CuO NCs on MCF7 breast cancer cells after 24 h of exposure (Figure 7). ZnO NPs exhibited relatively low capability in affecting MCF7 viability across the tested concentration range, with a statistically significant reduction observed only at higher concentrations (≥75 µg/mL). In contrast, CuO NPs had a pronounced effect on MCF7 cell viability, with a significant decrease already evident at 50 µg/mL, which became progressively stronger at higher concentrations, resulting in very low viability at 100–150 µg/mL. ZnO-CuO NCs demonstrated an intermediate profile, with a significant reduction in cell viability appearing from 75 µg/mL; however, the effect was less severe than that observed for CuO NPs and more pronounced than for ZnO NPs, suggesting that the combination of both oxides modulates the overall viability response. This concentration-dependent effect was further confirmed by IC_50_ determination, which revealed values of 103.43 µg/mL for ZnO NPs, 42.90 µg/mL for CuO NPs, and 71.12 µg/mL for ZnO-CuO NCs. Despite the use of similar synthesis routes, the NPs exhibited markedly different profiles on cell viability, which may be attributed to differences in their size and morphology. SEM and STEM analyses revealed pronounced differences in the morphology and size of CuO NPs, ZnO NPs, and ZnO-CuO NCs, regardless of the use of similar synthesis routes. It is worth highlighting that the hydrodynamic diameter of the NPs (357 nm for ZnO NPs, 174 nm for CuO NPs, and 248 nm for ZnO-CuO NCs) obtained from DLS is bigger than the estimated size from XRD results (~19 nm for ZnO NPs, ~8 nm for CuO NPs and ~13 nm for ZnO-CuO NCs) and SEM (~71 nm for ZnO NPs, ~38 nm for CuO NPs, and ~43 nm for ZnO-CuO NCs) and STEM (~214 nm for ZnO NPs, ~133 nm with very high polydispersity for CuO NPs, and about ~197 nm for ZnO-CuO NCs) because the technique provides the hydrodynamic diameter of NPs in suspension, which includes the solvation layer and particle agglomeration in the biological medium, rather than the actual size of individual NPs [57]. Interestingly, the DLS results for the three NPs are consistent with the SEM and TEM results, which provide more reliable information on the NPs’ morphology and size. Remarkably, the particle size distributions obtained across all techniques correlate well with the cytotoxicity profile, with CuO NPs, characterised by the smallest average size, exhibiting the highest effect on cell viability. In general, smaller NPs possess a higher surface-to-volume ratio, which enhances their chemical reactivity and promotes the generation of reactive oxygen species, ultimately leading to increased cellular damage [58,59]. Moreover, this trend is consistent with the crystallite sizes determined by XRD (Table 1), which were smallest for CuO NPs, further supporting the relationship between reduced particle/crystallite size and enhanced cytotoxic effects. Importantly, more detailed STEM images also revealed a fraction of very small CuO NPs (<20 nm), which, due to their exceptionally high surface reactivity and particularly efficient cellular internalisation, may substantially contribute to the pronounced cytotoxic effect observed.

**Figure 8:**
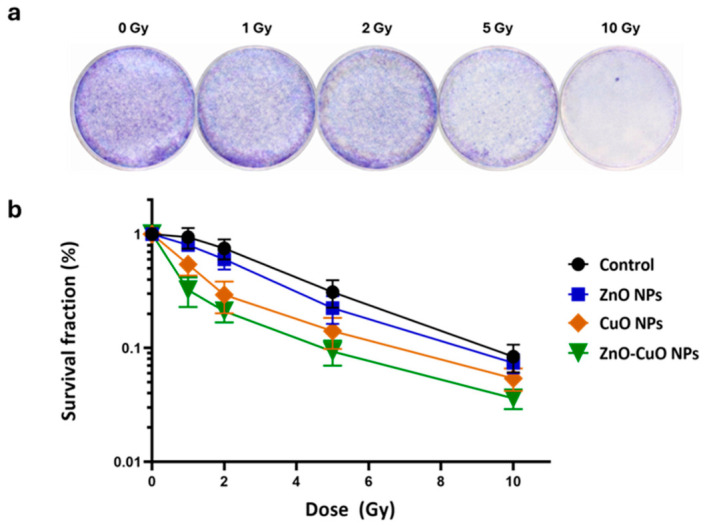
Representative images of MCF7 cell colonies obtained from the clonogenic assay under control conditions (0 Gy, without irradiation) and after proton beam irradiation at doses of 1, 2, 5, and 10 Gy, in the absence of NPs (**a**). Clonogenic survival curves of MCF7 cells following proton irradiation, in the presence or absence of NPs (**b**).

Furthermore, the radiosensitising properties of the synthesised NPs were investigated under simulated proton radiotherapy conditions using a clonogenic assay, the gold standard for assessing radiation-induced cellular damage. For this purpose, the same maximum non-toxic concentration (25 µg/mL) was used for all tested NPs types to ensure comparability of the results. This approach was particularly important given that radiosensitization does not necessarily scale linearly with the NPs’ incubation concentration, but rather with the actual degree of cellular metal internalisation, as reflected by changes in survival curve shape and enhancement factors reported previously [60]. Representative images of clonogenic colonies for the control group are shown in Figure 8a, while Figure 8b presents the dose-effect survival curves for proton beam irradiation in the absence or presence of ZnO NPs, CuO NPs, and ZnO-CuO NCs. Proton irradiation resulted in a dose-dependent decrease in clonogenic survival, which was further enhanced in the presence of NPs compared to the control. Among the tested materials, ZnO-CuO NCs exhibited the strongest radiosensitizing effect, followed by CuO NPs, while ZnO NPs showed the weakest enhancement of proton-induced cell killing, which could be due to their large size as compared to the others. The quantitative comparison of these effects as DEF values, defined as the ratio of the radiation dose required to achieve a given level of clonogenic cell survival in the control group to the dose required to achieve the same survival level in the presence of NPs, thereby reflecting the magnitude of NPs-induced radiosensitisation. Observed DEF values of 1.09 for ZnO NPs, 1.46 for CuO NPs, and 1.69 for ZnO-CuO NCs indicate a low, moderate, and strong radiosensitising effect, respectively. In the context of NPs-based radiosensitisation, a DEF close to 1.1 is generally considered modest, whereas values exceeding 1.4–1.5 are regarded as biologically relevant and indicative of a pronounced enhancement of radiation-induced cell killing, particularly under proton irradiation conditions. The results of the clonogenic assay indicate that the radiosensitising efficacy differs markedly among the investigated NPs, which is in good agreement with their morphological characteristics determined by STEM analysis. The strongest radiosensitising effects observed for ZnO-CuO NCs and CuO NPs can be attributed to their smaller effective sizes relative to ZnO NPs. NPs with smaller dimensions, characterised by a higher surface-to-volume ratio, exhibit increased surface reactivity, which may contribute to enhanced radiobiological effects following proton irradiation [61]. In contrast, the larger ZnO NPs showed the weakest radiosensitising effect, further highlighting the important role of NPs morphology in modulating the cellular response to radiation. In the context of proton therapy, it should be emphasised that the influence of NP size and the associated surface-to-volume ratio is less important for physical dose enhancement than in the case of photon irradiation. Nevertheless, these parameters may significantly modulate the radiobiological effect rather than the physical one, being associated with enhanced oxidative stress and altered cellular responses to radiation [62,63]. In the case of ZnO-CuO NCs, a synergistic effect from combining two metal oxides may also be considered, potentially leading to increased surface reactivity and more complex biological interactions, which could explain their markedly stronger radiosensitising effect compared to the individual components. This interpretation is further supported by earlier simulation studies on proton irradiation, which showed that bimetallic AuPt NCs exhibit a stronger radio-enhancing potential than monometallic Au NPs, suggesting that material complexity may contribute to amplified nano-mediated biological effects [64].

It should be emphasised that the biological experiments in the present study were performed using MCF7 breast cancer cells as the model tumour cell line. Therefore, can we guess which of the listed substances are capable of protecting cells from proton radiation? normal cells. However, previous studies on similar metal oxide NPs have shown lower toxicity toward normal cells than cancer cells. For example, spherical and rod-like TiO_2_ NPs were reported to exert stronger cytotoxic effects on SW480 colon cancer cells than on normal CRL-1790 cells, supporting the assumption that metal oxide-based NPs may display preferential toxicity toward cancer cells [65]. Similarly, Ag-doped TiO_2_ NPs were shown to be cytotoxic toward A549 lung cancer cells and MCF7 breast cancer cells, while exhibiting only limited toxicity toward normal hepatocytes and human lung fibroblasts [66]. Another example involves green-synthesised ZnO NPs obtained using *Vinca rosea* extract. These nanoparticles showed clear cytotoxicity toward MCF7 breast cancer cells and A549 lung cancer cells, with IC_50_ values of approximately 30 µg/mL, whereas treatment of normal MCF-10A breast epithelial cells with 60 µg/mL did not cause any noticeable decrease in cell viability [67]. However, to provide a balanced view, it should also be noted that selective toxicity is not observed for all metal oxide-based nanoparticles. For example, Fahmy et al. synthesised Cu/CuO NPs that exhibited comparable cytotoxic activity toward both cancerous A549 lung cancer cells and normal WI-38 lung normal cells [68]. This suggests that the NPs investigated in the present study may also exhibit a more favourable safety profile toward non-tumour cells, although this requires direct experimental confirmation.

### 3.8. Holotomographic Imaging of the Interactions Between MCF7 Cells and the Tested NPs

**Figure 9:**
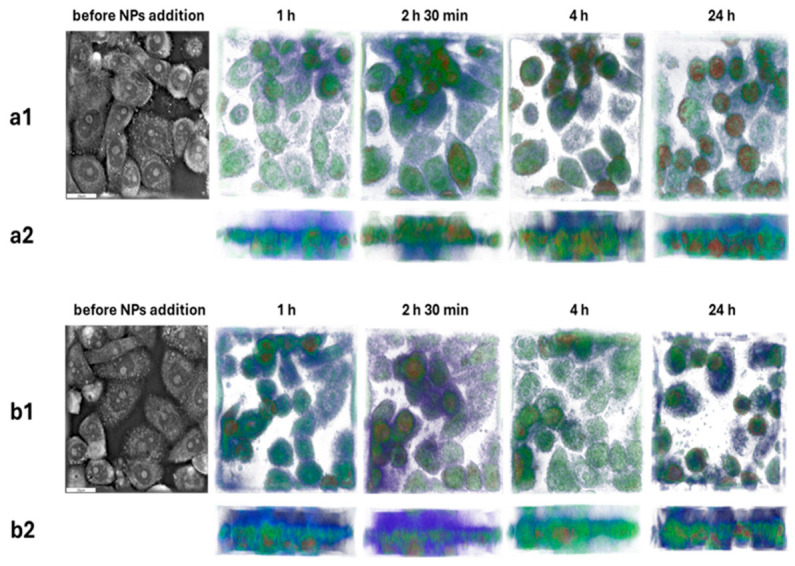
Initial 3D holotomographic image of MCF7 cells acquired using a Nanolive 3D microscope before ZnO NPs exposure is shown as a reference. Subsequently, 3D holotomographic images of MCF7 cells cultured with ZnO NPs (**a1**,**b1**), reconstructed based on refractive index (RI) values of ZnO NPs (red), nuclei and cytoplasm (green), and cell membrane (blue), are presented at different incubation time points after ZnO NPs addition. Z-axis reconstructions of the corresponding holotomographic images are shown in panels (**a2**,**b2**). The upper panels correspond to a ZnO NPs concentration of 25 µg/mL, while the lower panels correspond to 75 µg/mL.

**Figure 10:**
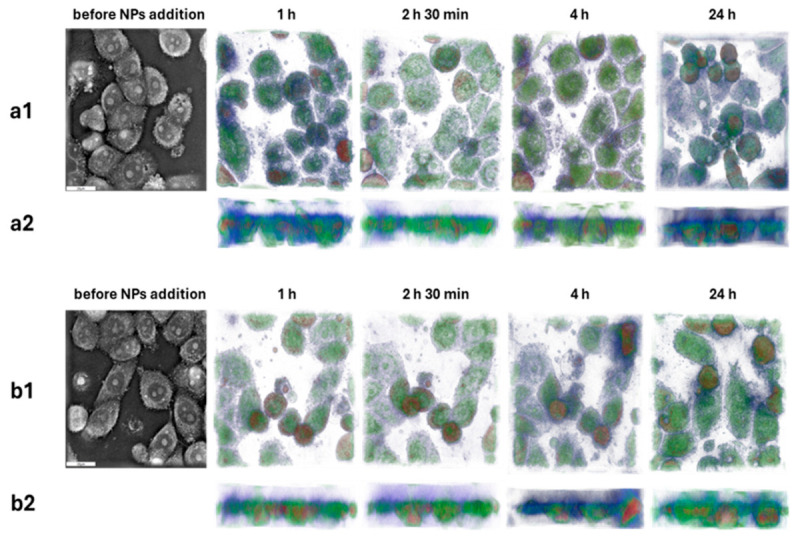
Initial 3D holotomographic image of MCF7 cells acquired using a Nanolive 3D microscope prior to CuO NPs exposure is shown as a reference. Subsequently, 3D holotomographic images of MCF7 cells cultured with CuO NPs (**a1**,**b1**), reconstructed based on refractive index (RI) values of CuO NPs (red), nuclei and cytoplasm (green), and cell membrane (blue), are presented at different incubation time points after CuO NPs addition. Z-axis reconstructions of the corresponding holotomographic images are shown in panels (**a2**,**b2**). The upper panels correspond to a CuO NPs concentration of 25 µg/mL, while the lower panels correspond to 75 µg/mL.

**Figure 11:**
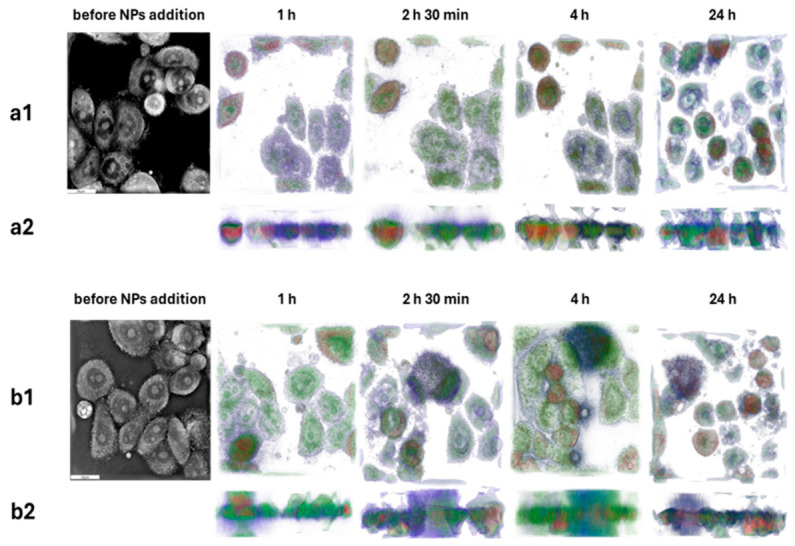
Initial 3D holotomographic image of MCF7 cells acquired using a Nanolive 3D microscope before ZnO-CuO NPs exposure is shown as a reference. Subsequently, 3D holotomographic images of MCF7 cells cultured with ZnO-CuO NCs (**a1**,**b1**), reconstructed based on refractive index (RI) values of ZnO-CuO NCs (red), nuclei and cytoplasm (green), and cell membrane (blue), are presented at different incubation time points after ZnO-CuO NCs addition. Z-axis reconstructions of the corresponding holotomographic images are shown in panels (**a2**,**b2**). The upper panels correspond to a ZnO-CuO NPs concentration of 25 µg/mL, while the lower panels correspond to 75 µg/mL.

The real interactions between the different types of NPs and MCF7 cells were investigated using holotomographic microscopy, which enables label-free, three-dimensional imaging of live cells and their responses to the presence of nanomaterials. Visualisation of NPs and individual cellular structures was possible due to differences in the refractive index (RI), which allows discrimination between cellular components (e.g., cytoplasm and cell membrane) and NPs. Based on these RI variations, three-dimensional holotomographic images of cells interacting with NPs were reconstructed. Figure 9, Figure 10 and Figure 11 present holotomographic 3D images acquired before and after NPs addition at different time points, together with Z-axis reconstructions, for ZnO NPs, CuO NPs, and ZnO-CuO NCs, respectively.

For ZnO NPs, no significant changes in cell morphology were observed at a concentration of 25 µg/mL (Figure 9(a1)), despite systematic cellular uptake of the NPs. Importantly, the number of cells within the field of view remained unchanged after 24 h, indicating relatively good cellular tolerance. In contrast, exposure to a higher ZnO NPs concentration of 75 µg/mL (Figure 9(b1)) partially inhibited long-term cell growth and led to pronounced cellular disintegration, as evidenced by the presence of numerous detached cellular fragments and debris after 24 h, suggesting loss of cellular integrity in a fraction of the population.

In the case of CuO NPs, no significant changes in cell morphology were observed at the early stages of exposure (1 h, 2 h 30 min, and 4 h) for either concentration, despite progressive CuO NPs uptake. After 24 h of incubation at a concentration of 25 µg/mL (Figure 10(a1)), the number of cells within the field of view remained comparable to the control. However, in a substantial fraction of the population, localised perforations of the cell membrane were evident, manifested as irregular “holes” within the cellular structure, which is in line with previous studies reported for other nanomaterials [69,70]. This phenomenon indicates a partial loss of membrane integrity while cell adhesion to the substrate was largely preserved. In contrast, at the higher CuO NPs concentration (Figure 10(b1)), no pronounced membrane perforations were observed. Instead, a subset of cells exhibited marked elongation; these cells contained relatively low amounts of internalised CuO NPs. Conversely, cells that accumulated substantial quantities of CuO NPs lost adhesion to the substrate, adopted a spherical morphology, and were observed as freely floating in the culture medium, indicating advanced functional and structural impairment. The absence of membrane perforation at the higher CuO NPs concentration may be explained by two possible factors. First, rapid and extensive NPs internalisation may activate alternative cell death pathways, resulting in loss of adhesion and cell rounding before localised membrane damage becomes morphologically apparent. Second, higher CuO NPs concentrations may induce acute oxidative stress and cytoskeletal disruption, which promote cell shape alterations and detachment rather than a gradual degradation of membrane integrity.

In the case of ZnO–CuO NCs, a distinct and intriguing response pattern was observed. At a concentration of 25 µg/mL (Figure 11(a1)), a time-dependent increase in NPs uptake was recorded, accompanied by relatively minor changes in cell morphology throughout the observation period. Cells largely preserved their original shape and adhesion, indicating a comparatively moderate cellular response despite progressive internalisation of the NPs. In contrast, exposure to ZnO–CuO NCs at the higher concentration of 75 µg/mL (Figure 11(b1)) induced pronounced morphological alterations in a subset of cells. These changes were characterised by progressive cell stretching and apparent swelling, suggestive of transient cellular expansion or cytoskeletal remodelling. The phenomenon of cell swelling induced by nanoparticles has also been reported previously in the literature, including in the context of protein- and lipid-based nanomaterials [71,72]. Notably, after 24 h of incubation, a fraction of the affected cells partially reverted to their original morphology. However, this recovery-like behaviour was accompanied by the presence of visible cellular fragments within the field of view, indicating that the process was incomplete and that structural damage or loss of cellular material had occurred in part of the population.

To confirm that all tested NPs were internalised rather than merely adsorbed onto the outer cell membrane, Z-axis reconstructions were performed, clearly demonstrating the presence of NPs within the cellular volume, as shown in Figure 9, Figure 10 and Figure 11 (panels a2 and b2 in each figure). Furthermore, to better visualise the spatial localisation and accumulation sites of the NPs, supplementary videos were generated showing 3D reconstructions after 4 h of incubation of MCF7 cells with the individual types of NPs at different concentrations (Movie S1–Movie S6). Overall, the tested NPs were predominantly distributed in the vicinity of the outer cell membrane, while a fraction of them also appeared to be more randomly dispersed within the cytoplasm. This general distribution pattern was observed for most of the investigated nanomaterials. Interestingly, a distinct localisation pattern was witnessed in some cells at high concentrations of ZnO–CuO NCs. In these cases, NPs showed punctate accumulation in specific regions of the cytoplasm, possibly in perinuclear areas or near mitochondria. Due to the relatively small differences in RI between these organelles, their unambiguous discrimination by holotomographic microscopy is challenging. Nevertheless, such localised intracellular clustering of NPs was clearly observable in the 3D reconstructions. Such a localisation pattern may partially explain the previously observed superior radiosensitising potential of these ZnO–CuO NCs, as NPs located closer to cellular DNA have been shown to more effectively enhance radiation-induced effects, including increased local energy deposition and amplification of DNA damage [73,74,75,76,77].

**Figure 12:**
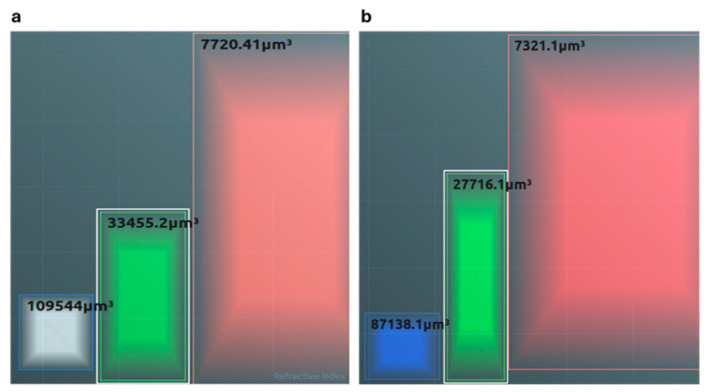
Exemplary distribution of RI with the value of RI volume corresponding to individual structures: ZnO NPs (red), nuclei and cytoplasm (green), and cell membrane (blue) for MCF7 cells incubated with ZnO NPs for 4 h at concentrations of 25 µg/mL (**a**) and 75 µg/mL (**b**).

Finally, according to the holotomographic images, the distribution of RI values was determined, with RI volume ranges assigned to individual cellular structures, including NPs, nuclei/cytoplasm, and the cell membrane. Representative examples of the obtained RI distributions are presented in Figure 12. Based on the RI distributions obtained at different time points, the intracellular volume occupied by NPs was quantitatively estimated. This was achieved by calculating the percentage ratio of the volume attributed to NPs to the total cellular volume, defined as the sum of the cytoplasmic and cell membrane volumes. This normalised approach was considered more appropriate than directly comparing absolute NPs volumes expressed in µm^3^. During the experiment, particularly at longer incubation times, dynamic changes in the cell population were observed, including cell division, loss of adhesion, and cells exiting the field of view. As a result, absolute NPs volume values could be biased by these effects. In contrast, the use of a relative, percentage-based metric provides a more robust and reliable quantitative representation of cellular NPs uptake over time at the single-cell level. The corresponding values obtained using this approach are summarised in Table 3 for all investigated NP types and the different incubation times with MCF7 cells.

## 4. Conclusions

The present study used an eco-friendly approach to synthesised ZnO NPs, CuO NPs, and ZnO-CuO NCs with *S. africana* Luteus. Their anticancer activity against the MCF7 breast cancer cell line was examined, along with their radiosensitising effect in radiotherapy. The nanoparticles were found to be spherical to irregular, pure, and crystalline, with a similar particle-size distribution obtained from DLS, SEM, and TEM. All nanoparticles showed dose-dependent cytotoxic effects on MCF7 cells, with CuO NPs exhibiting the strongest effect, followed by ZnO-CuO NCs and ZnO NPs. Additionally, clonogenic survival assays, considered the gold standard for evaluating radiation-induced cell damage, demonstrated that all nanoparticles enhanced proton-induced cell death in a dose-dependent manner. Among them, ZnO-CuO NCs had the highest radiosensitising effect, linked to increased local energy deposition and greater DNA damage, followed by CuO NPs and ZnO NPs. Interactions between nanoparticles and MCF7 cells observed through holotomographic imaging confirmed that ZnO-CuO NCs caused significantly more damage to cellular macromolecules than the others, likely due to the synergistic effect of combining two metal oxides. This combination increases surface reactivity and the production of reactive oxygen species, leading to greater oxidative stress and cellular damage. Overall, biogenic ZnO-CuO NCs outperform single-metal oxides as radiosensitizers due to their synergistic effects, enhanced cellular uptake, and increased ROS generation. These findings highlight the potential of ZnO-CuO NCs synthesised with *S. africana* Luteus as innovative radiosensitizers in cancer radiotherapy. Future studies should explore the in vitro and in vivo efficacy of the synthesised nanostructures and provide a more comprehensive understanding of the molecular mechanisms underlying their biological and radiosensitising activities. In particular, investigations of ROS generation, DNA damage, γ-H2AX expression, comet assay, apoptosis and necrosis pathways, cell cycle alterations, mitochondrial dysfunction, and oxidative stress responses will be important to clarify the mechanisms responsible for the enhanced anticancer and radiosensitising effects observed in this study. Furthermore, our results could aid computational biologists in developing biologically based treatment plans for cancer radiotherapy. This research could pave the way for the development of next-generation radiosensitizers produced through sustainable, safe, and more effective methods for cancer treatment.

## Figures and Tables

**Table 1:** The hydrodynamic diameter (DLS), polydispersity index (PDI), zeta potential, and TEM of ZnO NPs, CuO NPs, and ZnO-CuO NCs synthesised using *S. africana* Luteus.

Parameters	ZnO NPs	CuO NPs	ZnO-CuO NPs
Hydrodynamic diameter (DLS) (nm)	357	174	248
Polydispersity index (PDI)	0.36	0.48	0.22
Zeta potential (mV)	−30.78	−11.31	−18.64
TEM (nm)	~214	~133	~197

**Table 2:** Average crystallite size of ZnO NPs, CuO NPs, and ZnO-CuO NCs synthesised using aqueous extract of *S. africana* Luteus.

Sample	Peak Position	FWHM	Crystallite Size	Average Size
ZnO NPs	31.72911	0.45265	19.05650011	18.54 ± 5.26
	34.37569	0.28346	30.64009777	
	36.218	0.46687	18.69852742	
	47.49188	0.68058	13.31921865	
	56.55754	0.53182	17.71621391	
	62.81131	0.59315	16.38976172	
	67.91321	0.5642	17.73010879	
	69.03413	0.68024	14.80380742	
CuO NPs	35.47973	0.9593	9.081213379	7.89 ± 1.90
	38.64489	1.04947	8.378177486	
	48.76868	0.9553	9.536285215	
	53.09225	7.49797	1.237029305	
	58.35061	2.2442	4.234467294	
	61.54766	1.82393	5.294697307	
	66.2536	3.69564	2.68092704	
	68.0371	0.72402	13.82644328	
ZnO-CuO NCs	31.77512	0.9574	9.010768592	12.91 ± 5.96
	34.72433	1.79174	4.85196718	
	35.45778	0.39454	22.07906513	
	36.19822	1.02847	8.487645602	
	38.67107	0.82938	10.60231808	
	47.565	0.92695	9.781909486	
	48.76422	0.68478	13.30332742	
	53.49784	0.35101	26.47131681	
	56.60372	0.77666	12.13385617	
	58.14331	0.67352	14.09522891	
	61.55503	0.65494	14.74566702	
	62.84849	1.04638	9.292526462	
	66.18225	1.27908	7.742845729	
	67.97759	0.97383	10.27603893	
	69.07393	0.48525	20.7574415	

**Table 3:** Ratios of the volume occupied by individual NPs within MCF7 cells relative to the total cell volume, expressed as percentages, at different NPs incubation times, calculated based on 3D holotomographic imaging.

Sample Concentration	1 h	2 h 30 min	4 h	24 h
ZnO NPs 25 µg/mL	0.56	1.94	5.40	5.33
ZnO NPs 75 µg/mL	1.12	3.23	6.37	5.12
CuO NPs 25 µg/mL	2.24	3.54	3.97	4.43
CuO NPs 75 µg/mL	2.89	3.78	4.98	5.65
ZnO-CuO NCs 25 µg/mL	0.93	2.94	3.73	4.41
ZnO-CuO NCs 75 µg/mL	4.34	7.89	9.69	9.43

## Data Availability

Data will be available upon request from the corresponding author.

## Supplementary Materials

The following supporting information can be downloaded at: https://www.mdpi.com/article/10.3390/nano16130789/s1, Movie S1: 3D reconstruction of a holotomographic image showing MCF7 cells after 4 h of incubation with ZnO NPs at a concentration of 25 µg/mL; Movie S2: 3D reconstruction of a holotomographic image showing MCF7 cells after 4 h of incubation with ZnO NPs at a concentration of 75 µg/mL; Movie S3: 3D reconstruction of a holotomographic image showing MCF7 cells after 4 h of incubation with CuO NPs at a concentration of 25 µg/mL; Movie S4: 3D reconstruction of a holotomographic image showing MCF7 cells after 4 h of incubation with CuO NPs at a concentration of 75 µg/mL; Movie S5: 3D reconstruction of a holotomographic image showing MCF7 cells after 4 h of incubation with ZnO-CuO NPs at a concentration of 25 µg/mL; Movie S6: 3D reconstruction of a holotomographic image showing MCF7 cells after 4 h of incubation with ZnO-CuO NPs at a concentration of 75 µg/mL.
