# Supplementary material for: Biogenic ZnO-CuO Nanocomposites Synthesised Using Salvia africana Luteus Increased the Radiosensitising Effect of Proton Irradiation in MCF7 Breast Cancer Cells
Source: Nanomaterials (Basel). 2026 Jun 23;16(13):789. doi: 10.3390/nano16130789 (PMC13362710; doi:10.3390/nano16130789)
Supplement: Supplementary file 1 [file nanomaterials-16-00789-s001.zip › Supplementary information_nanomaterils.pdf]

Supporting Information

Biogenic ZnO-CuO nanocomposites synthesized using *Salvia africana* Luteus increased the radiosensitizing effect of proton irradiation in MCF7 breast cancer cells

Kunle Okaiyeto[1], Bartosz Klebowski[2], Susi Zara[1], Maria Rosa Gigliobianco[1], and Piera Di Martino[1],*

**Corresponding Author: maria.gigliobianco@unich.it*

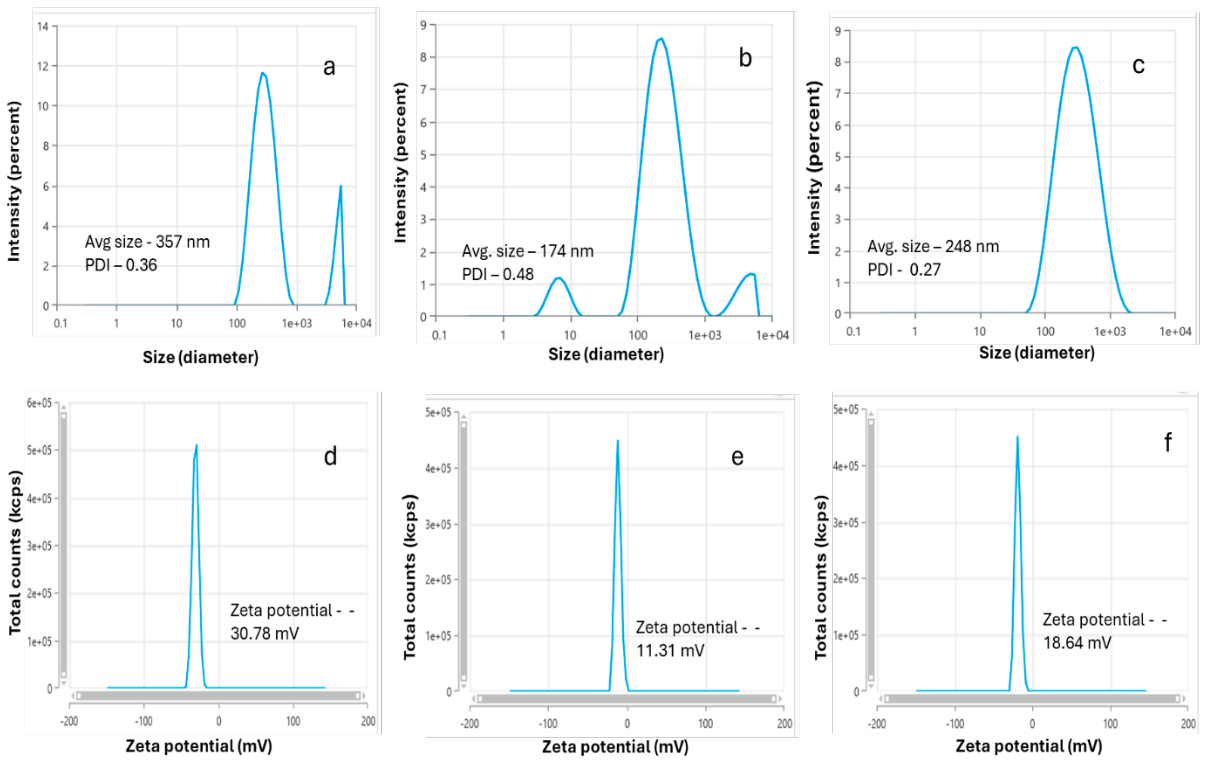

Figure S1: DLS analysis of ZnO NPs (a), CuO NPs (b), and ZnO-CuO NCs synthesized using *S. africana* Luteus. Zeta potential of ZnO NPs (d), CuO NPs (e), and ZnO-CuO NCs (f) synthesized using *S. africana* Luteus.
